# Supplementary material for: MTCH2 controls energy demand and expenditure to fuel anabolism during adipogenesis
Source: EMBO J. 2025 Jan 3;44(4):1007–38. doi: 10.1038/s44318-024-00335-7 (PMC11832942; doi:10.1038/s44318-024-00335-7)
Supplement: Supplementary file 5 — Source data Fig. 1 [file 44318_2024_335_MOESM5_ESM.zip › Figure1/1C .pdf]

Figure 1C

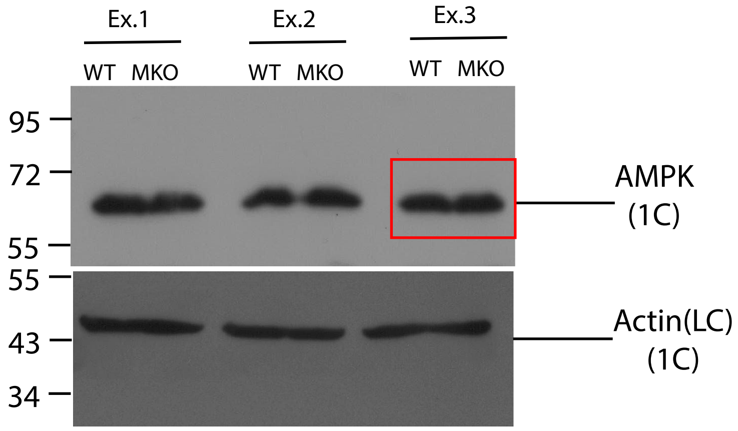

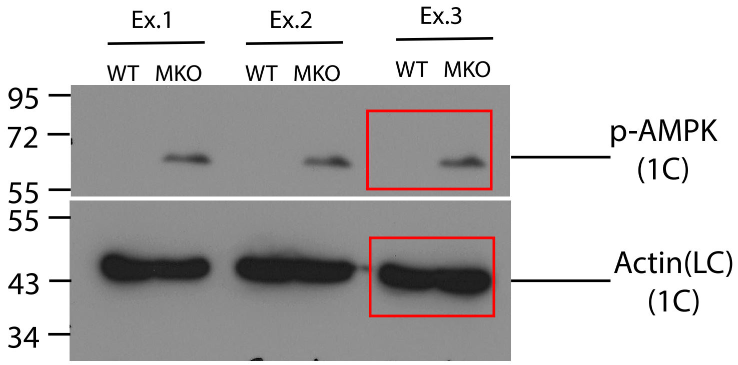

Figure1C. Cropped area of westren blot used in Fig.1C are in red. Blots are cut and probed for different primary antibodies(target protein and its loading control). Blots are developed on X-ray films at different exposure time.
