# Supplementary figures and images for: MTCH2 controls energy demand and expenditure to fuel anabolism during adipogenesis
Source: EMBO J. 2025 Jan 3;44(4):1007–38. doi: 10.1038/s44318-024-00335-7 (PMC11832942; doi:10.1038/s44318-024-00335-7)

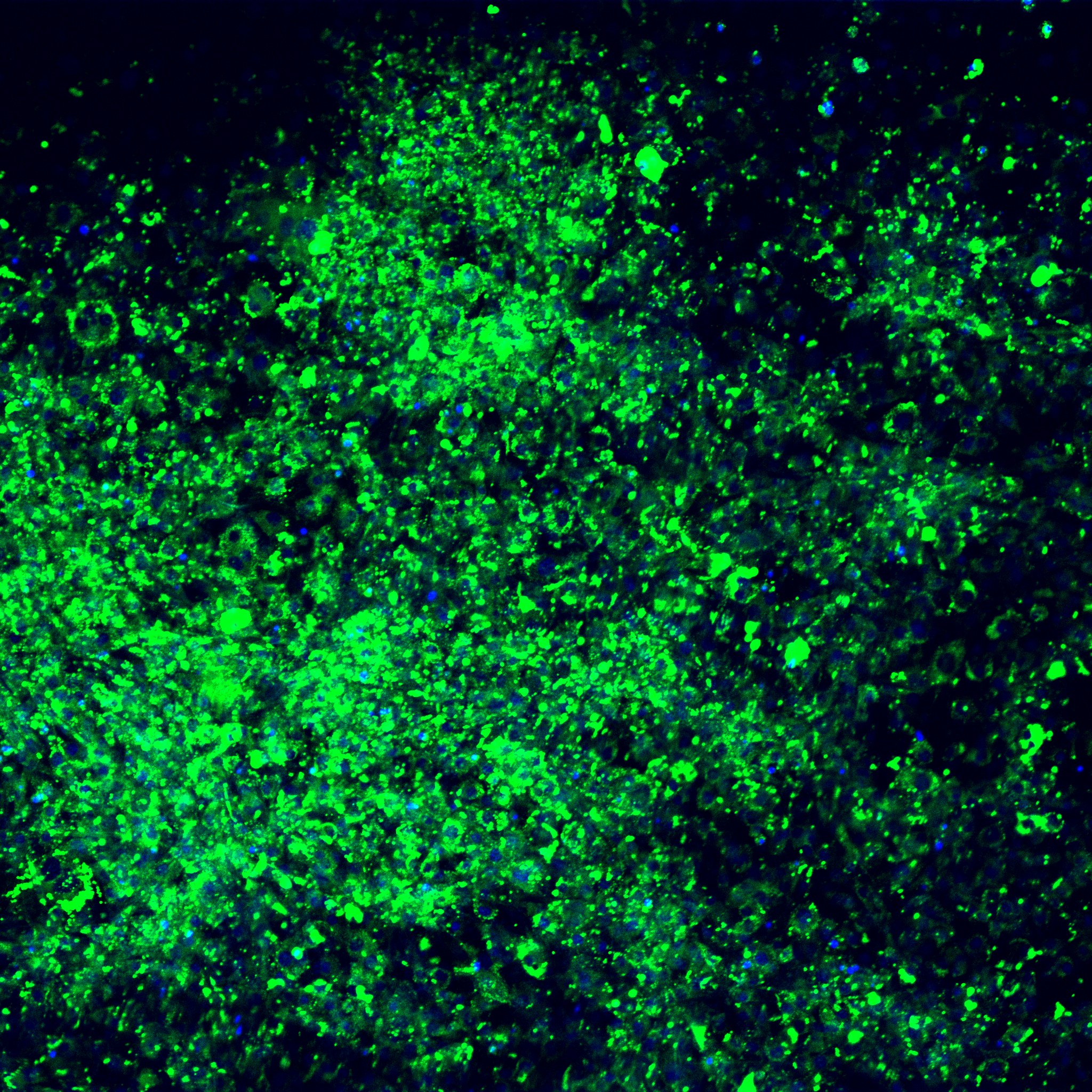

Supplement: Supplementary file 9 — Source data Fig. 5 [file 44318_2024_335_MOESM9_ESM.zip › Figure5/5A/NIH3T3L1_KO_D6_10x.tif]

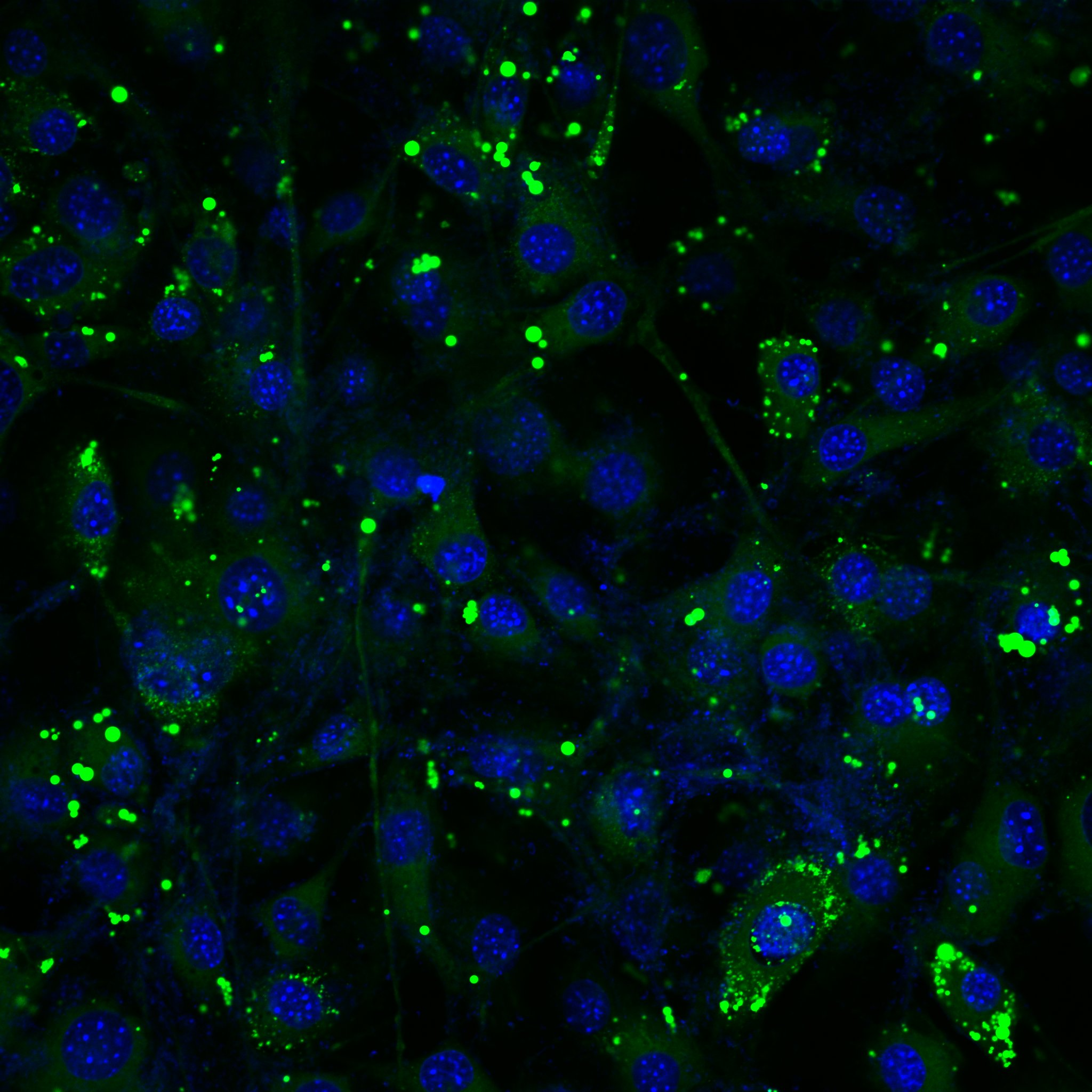

Supplement: Supplementary file 9 — Source data Fig. 5 [file 44318_2024_335_MOESM9_ESM.zip › Figure5/5A/NIH3T3L1_KO_D6_40x.tif]

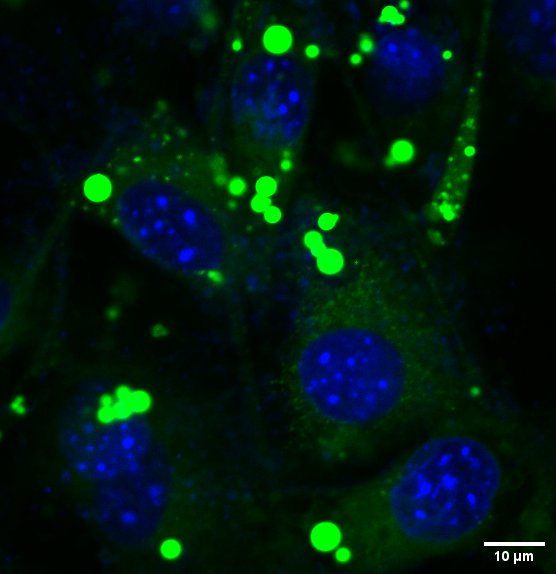

Supplement: Supplementary file 9 — Source data Fig. 5 [file 44318_2024_335_MOESM9_ESM.zip › Figure5/5A/NIH3T3L1_KO_D6_Inset.tif]

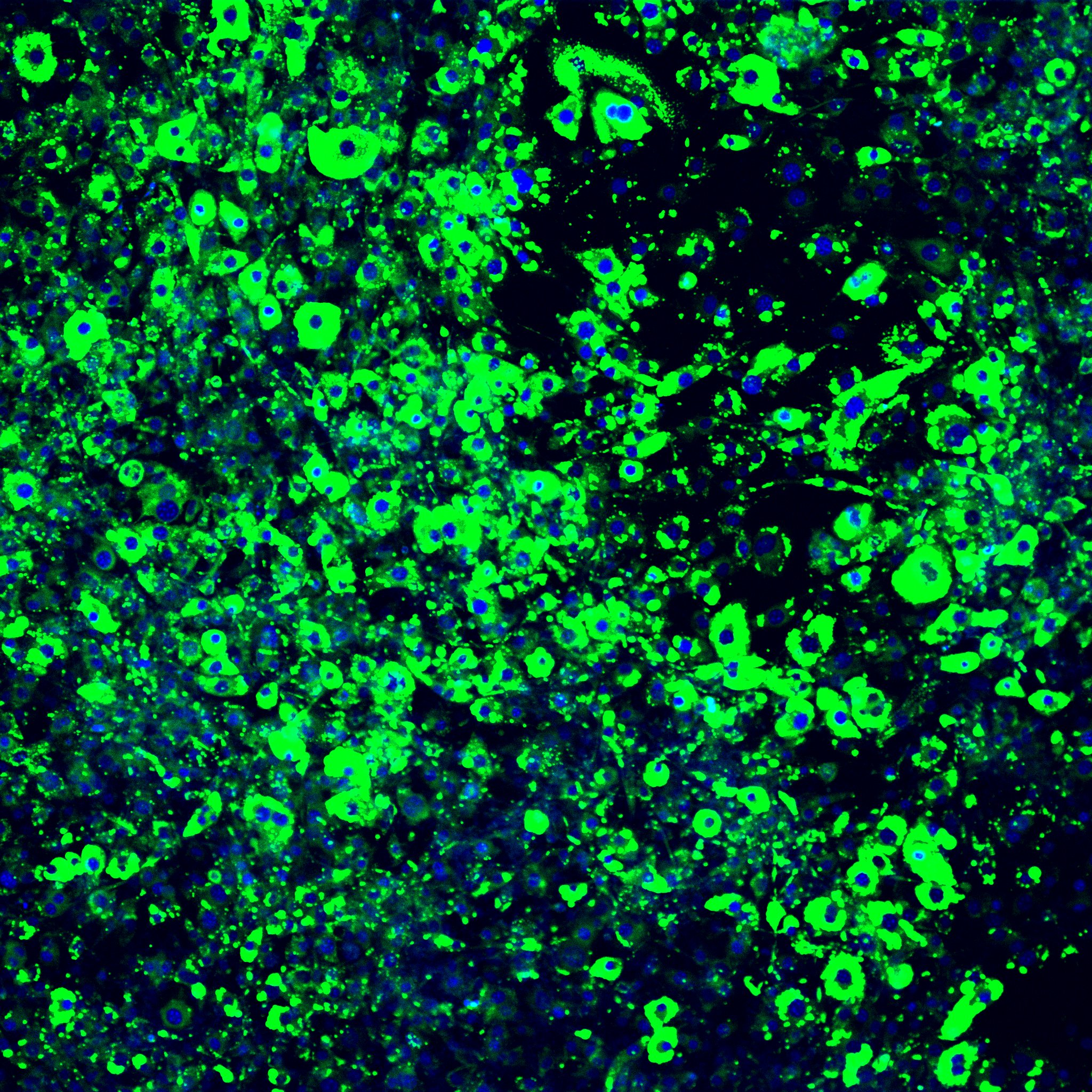

Supplement: Supplementary file 9 — Source data Fig. 5 [file 44318_2024_335_MOESM9_ESM.zip › Figure5/5A/NIH3T3L1_WT__D6_10x.tif]

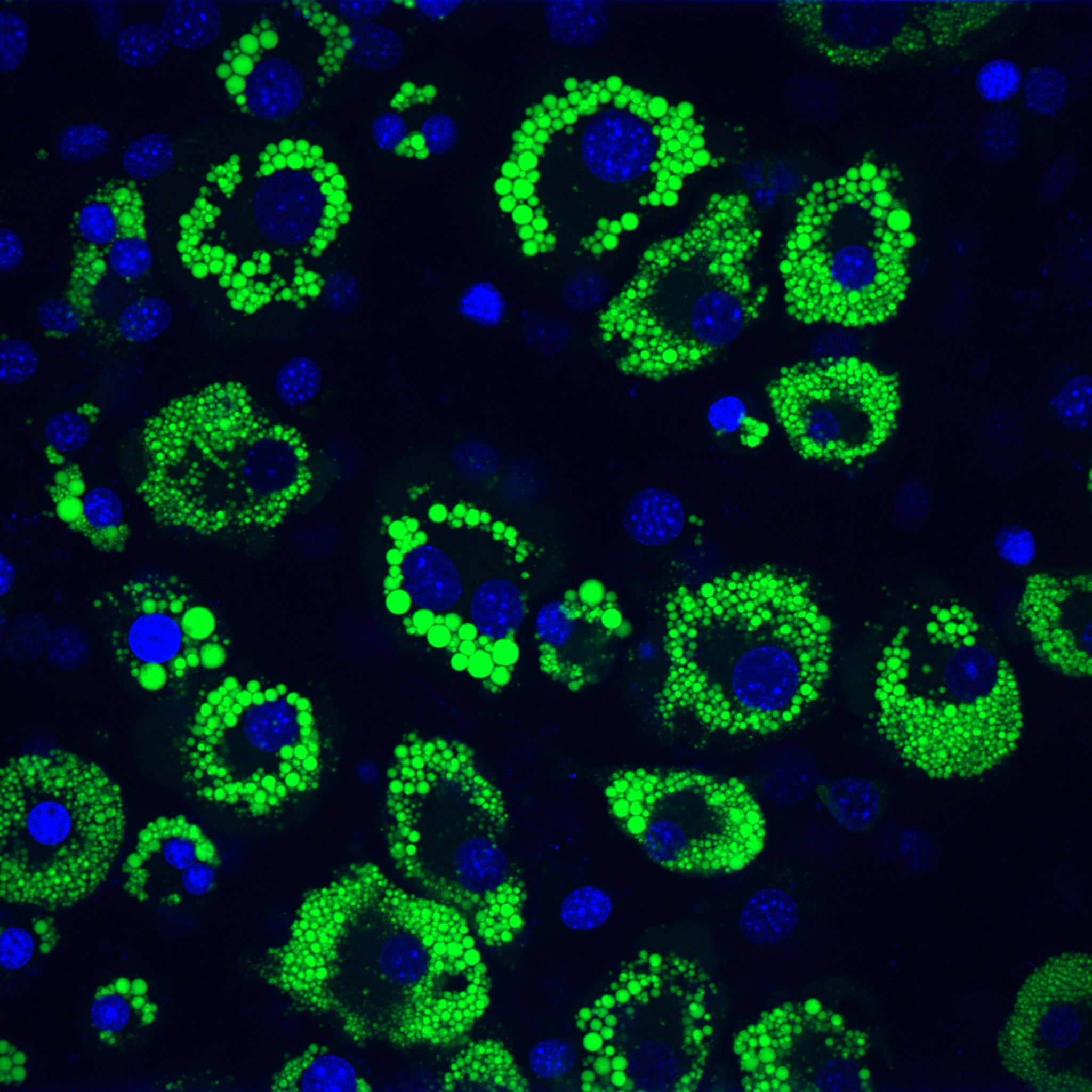

Supplement: Supplementary file 9 — Source data Fig. 5 [file 44318_2024_335_MOESM9_ESM.zip › Figure5/5A/NIH3T3L1_WT_D6_40x.tif]

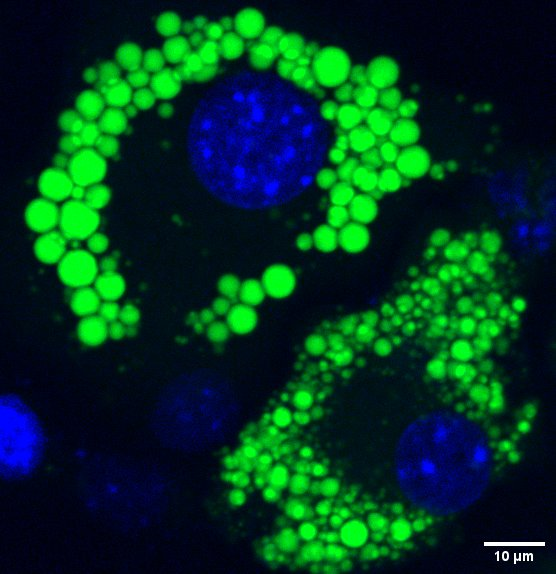

Supplement: Supplementary file 9 — Source data Fig. 5 [file 44318_2024_335_MOESM9_ESM.zip › Figure5/5A/NIH3T3L1_WT_D6_Inset.tif]
