# Supplementary material for: MTCH2 controls energy demand and expenditure to fuel anabolism during adipogenesis
Source: EMBO J. 2025 Jan 3;44(4):1007–38. doi: 10.1038/s44318-024-00335-7 (PMC11832942; doi:10.1038/s44318-024-00335-7)
Supplement: Supplementary file 11 — Expanded View Figures [file 44318_2024_335_MOESM11_ESM.pdf]

Expanded View Figures

Figure EV1. Global untargeted metabolomics analysis of WT, MKO, and MKO-R cells.

(**A**) MTCH2 mRNA expression checked by RT-PCR in all 6 clones of WT and MKO and WT cells. Results are presented as mean ± SEM (****$P<0.0007$, ordinary one-way ANOVA). N=Two independent experiments. (**B**) A representative immunoblot of MTCH2 protein level by for MTCH2 expression in all 6 clones of MKO and WT cells. (**C**) A heat map comparing the levels of the top 70 metabolites (out of 107 differential metabolites detected in global metabolomics, all 107 differential metabolites appear in Dataset EV1) in the WT, MKO, and MKO-R cell lines. Metabolite concentration values (Relative abundance) were log 1.5-transformed for statistics. The groups were compared by ANOVA. Values are scaled to Z-scores per row (metabolites); $n=4$ independent biological replicates. (**D**) Pathway enrichment analysis of 107 differential metabolites detected in global metabolomics. Enrichment was detected using a Hypergeometric Test using a Relative-betweenness Certrality topology against the Homo sapiens (KEGG) database, using MetaboAnalyst server ($n=4$ independent biological replicates). (**E**) Number of metabolites detected in significantly enriched pathways with an FDR cutoff<0.12 ($n=4$ independent biological replicates). Source data are available online for this figure.

© The Author(s)

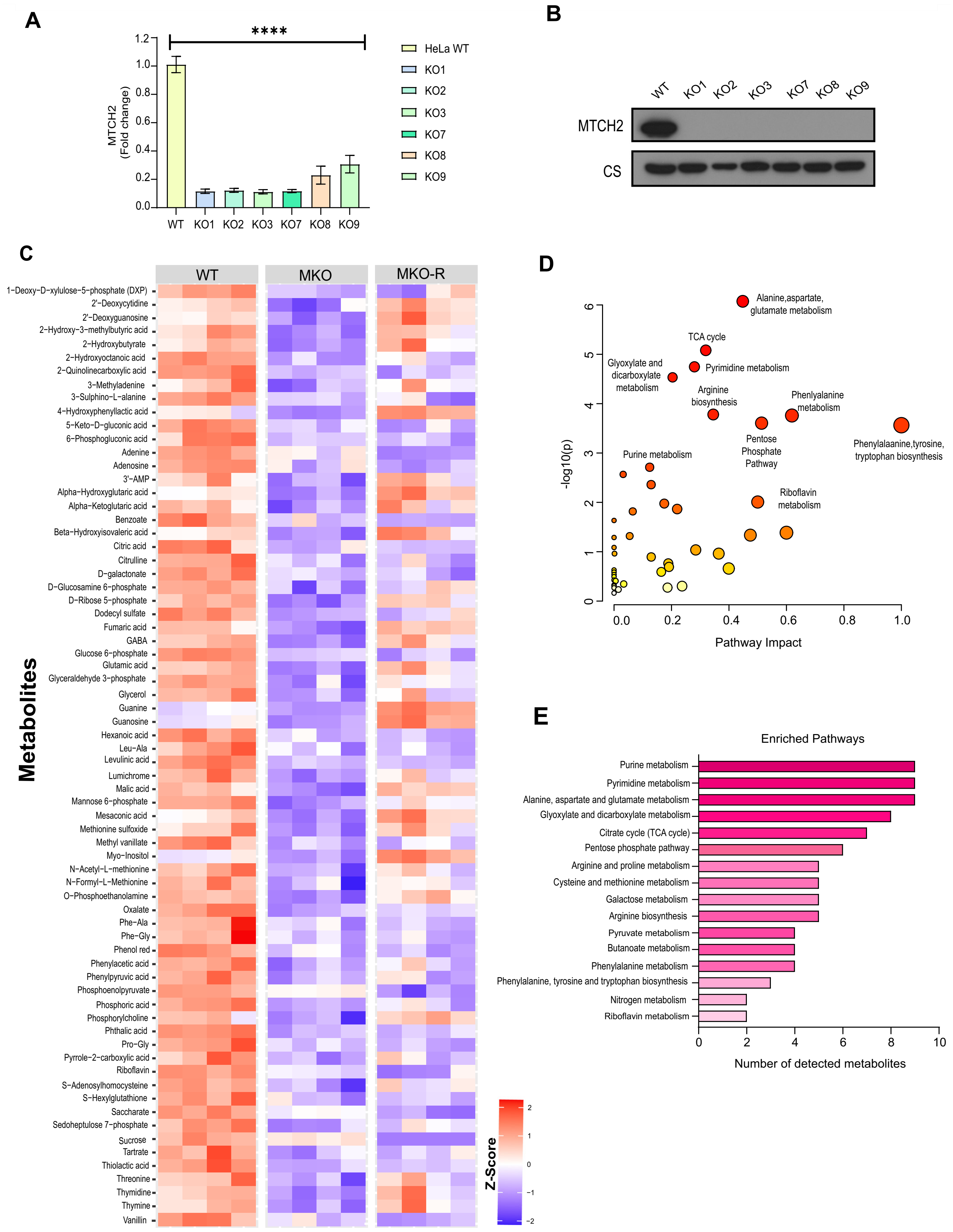
A
****
MTCH2 (Fold change)
1.2
1.0
0.8
0.6
0.4
0.2
0.0
WT KO1 KO2 KO3 KO7 KO8 KO9
HeLa WT
KO1
KO2
KO3
KO7
KO8
KO9
B
WT KO1 KO2 KO3 KO7 KO8 KO9
MTCH2
CS
C
WT
MKO
MKO-R
Metabolites
1-Deoxy-D-xylulose-5-phosphate (DXP)
2'-Deoxycytidine
2'-Deoxyguanosine
2-Hydroxy-3-methylbutyric acid
2-Hydroxybutyrate
2-Hydroxyoctanoic acid
2-Quinolinecarboxylic acid
3-Methyladenine
3-Sulphino-L-alanine
4-Hydroxyphenyllactic acid
5-Keto-D-gluconic acid
6-Phosphogluconic acid
Adenine
Adenosine
3'-AMP
Alpha-Hydroxyglutaric acid
Alpha-Ketoglutaric acid
Benzoate
Beta-Hydroxyisovaleric acid
Citric acid
Citrulline
D-galactonate
D-Glucosamine 6-phosphate
D-Ribose 5-phosphate
Dodecyl sulfate
Fumaric acid
GABA
Glucose 6-phosphate
Glutamic acid
Glyceraldehyde 3-phosphate
Glycerol
Guanine
Guanosine
Hexanoic acid
Leu-Ala
Levulinic acid
Lumichrome
Malic acid
Mannose 6-phosphate
Mesaconic acid
Methionine sulfoxide
Methyl vanillate
Myo-Inositol
N-Acetyl-L-methionine
N-Formyl-L-Methionine
O-Phosphoethanolamine
Oxalate
Phe-Ala
Phe-Gly
Phenol red
Phenylacetic acid
Phenylpyruvic acid
Phosphoenolpyruvate
Phosphoric acid
Phosphorylcholine
Phthalic acid
Pro-Gly
Pyrrole-2-carboxylic acid
Riboflavin
S-Adenosylhomocysteine
S-Hexylglutathione
Saccharate
Sedoheptulose 7-phosphate
Sucrose
Tartrate
Thiolactic acid
Threonine
Thymidine
Thymine
Vanillin
Z-Score
2
1
0
-1
-2
D
Alanine,aspartate, glutamate metabolism
TCA cycle
Glyoxylate and dicarboxylate metabolism
Pyrimidine metabolism
Arginine biosynthesis
Phenlyalanine metabolism
Pentose Phosphate Pathway
Phenylalaanine,tyrosine, tryptophan biosynthesis
Purine metabolism
Riboflavin metabolism
-log10(p)
0
1
2
3
4
5
6
0.0
0.2
0.4
0.6
0.8
1.0
Pathway Impact
E
Enriched Pathways
Purine metabolism
Pyrimidine metabolism
Alanine, aspartate and glutamate metabolism
Glyoxylate and dicarboxylate metabolism
Citrate cycle (TCA cycle)
Pentose phosphate pathway
Arginine and proline metabolism
Cysteine and methionine metabolism
Galactose metabolism
Arginine biosynthesis
Pyruvate metabolism
Butanoate metabolism
Phenylalanine metabolism
Phenylalanine, tyrosine and tryptophan biosynthesis
Nitrogen metabolism
Riboflavin metabolism
0
2
4
6
8
10
Number of detected metabolites

 © The Author(s)

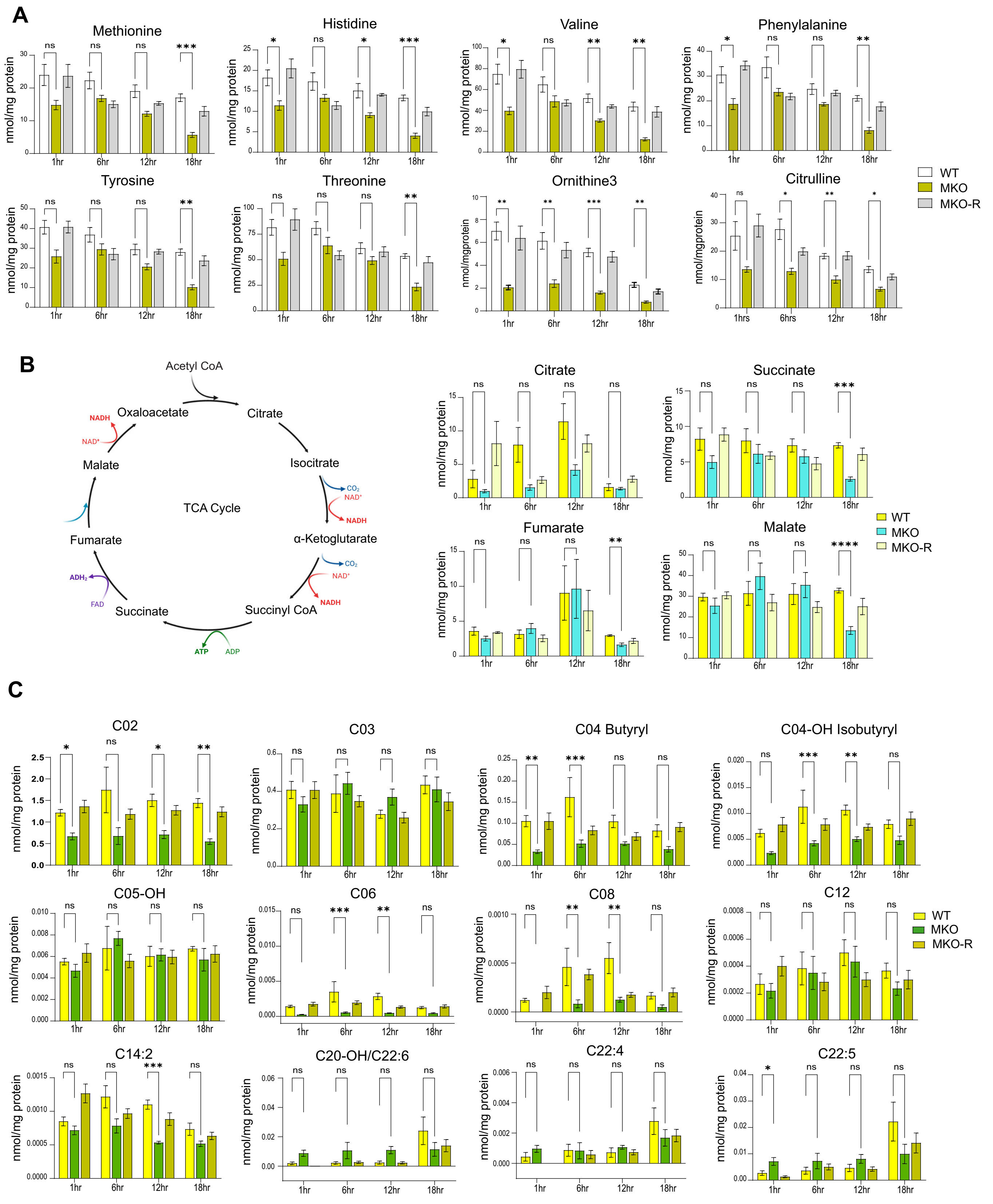
A
Methionine
Histidine
Valine
Phenylalanine
Tyrosine
Threonine
Ornithine3
Citrulline
nmol/mg protein
1hr
6hr
12hr
18hr
WT
MKO
MKO-R
B
Acetyl CoA
Citrate
Isocitrate
α-Ketoglutarate
Succinyl CoA
Succinate
Fumarate
Malate
Oxaloacetate
TCA Cycle
NADH
NAD+
CO2
ATP
ADP
FAD
ADH2
Citrate
Succinate
Fumarate
Malate
C
C02
C03
C04 Butyryl
C04-OH Isobutyryl
C05-OH
C06
C08
C12
C14:2
C20-OH/C22:6
C22:4
C22:5

◀ **Figure EV2. An increase in amino acid/TCA cycle/lipid utilization in MKO cells.**

(**A**) Average levels of a set of amino acids in all 3 cell lines at all four time points. (**B**) Left panel: Schematic representation of the TCA cycle. Right panels: Average levels of a set of TCA cycle intermediates in all 3 cell lines at all four time points. (**C**) Average levels of acylcarnitines in all 3 cell lines at all four time points. Results in all graphs in (**A**–**C**) are presented as mean ± SEM (ns, non-significant, **P*<0.05, ***P*<0.001, ****P*<0.0003, *****P*<0.0007; two-way ANOVA with Dunnett multiple comparison test; *n*=6 biological replicates). Source data are available online for this figure.

 © The Author(s)

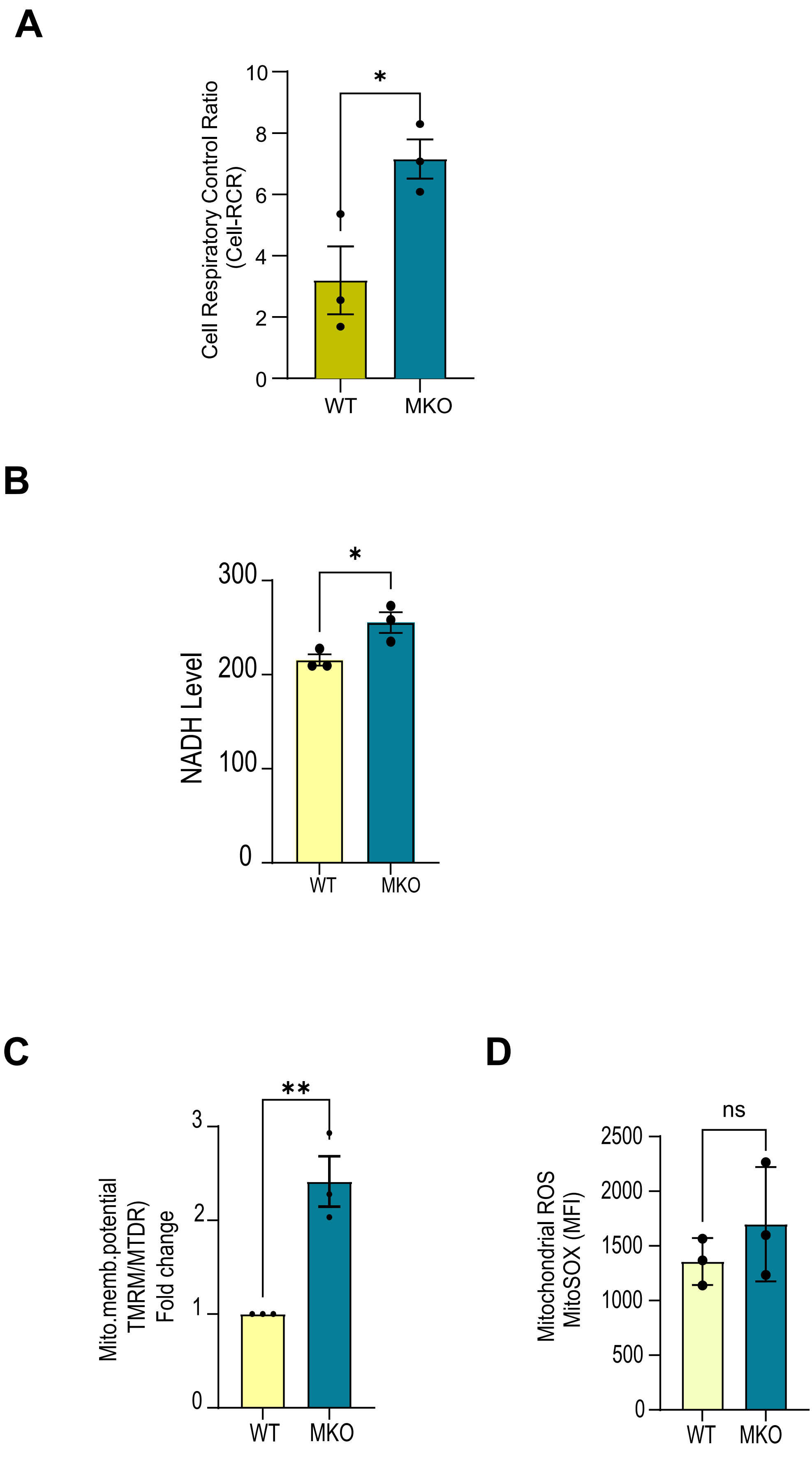
A
*
Cell Respiratory Control Ratio (Cell-RCR)
10
8
6
4
2
0
WT
MKO
B
*
NADH Level
300
200
100
0
WT
MKO
C
**
Mito.memb.potential TMRM/MTDR) Fold change
3
2
1
0
WT
MKO
D
ns
Mitochondrial ROS MitoSOX (MFI)
2500
2000
1500
1000
500
0
WT
MKO

Figure EV3. Increased mitochondrial oxidative function in MKO cells.

(**A**) Cell Respiratory Control Ratio of WT and MKO cells. Results are presented as mean ± SEM (Unpaired *t* test, **P*<0.05. *N*=3 independent experiments with 6–8 technical replicates in each experiment). (**B**) Increased total mitochondrial NADH content in MKO cells. Mitochondrial NADH levels were calculated as described in the Methods. Results are presented as mean ± SEM (Unpaired *t* test, **P*<0.05, *N*=3 independent experiments). Mitochondrial NADH content was calculated as the difference in NADH mean autofluorescence intensity (MFI). Maximal NADH autofluorescence was determined in response to KCN and minimal NADH autofluorescence was determined in response to FCCP as described in the Methods. (**C**) Increased mitochondrial membrane potential in MKO cells. Results are presented as means ± SEM (Unpaired *t* test, ***P*<0.001, *N*=3 independent experiments). (**D**) Mitochondrial ROS levels in WT and MKO cells. Levels of mitochondrial ROS (measured using mitoSOX) are presented. Results are presented as mean ± SEM (Unpaired *t* test, ns-nonsignificant, *N*=3 independent experiments). Source data are available online for this figure.

 © The Author(s)

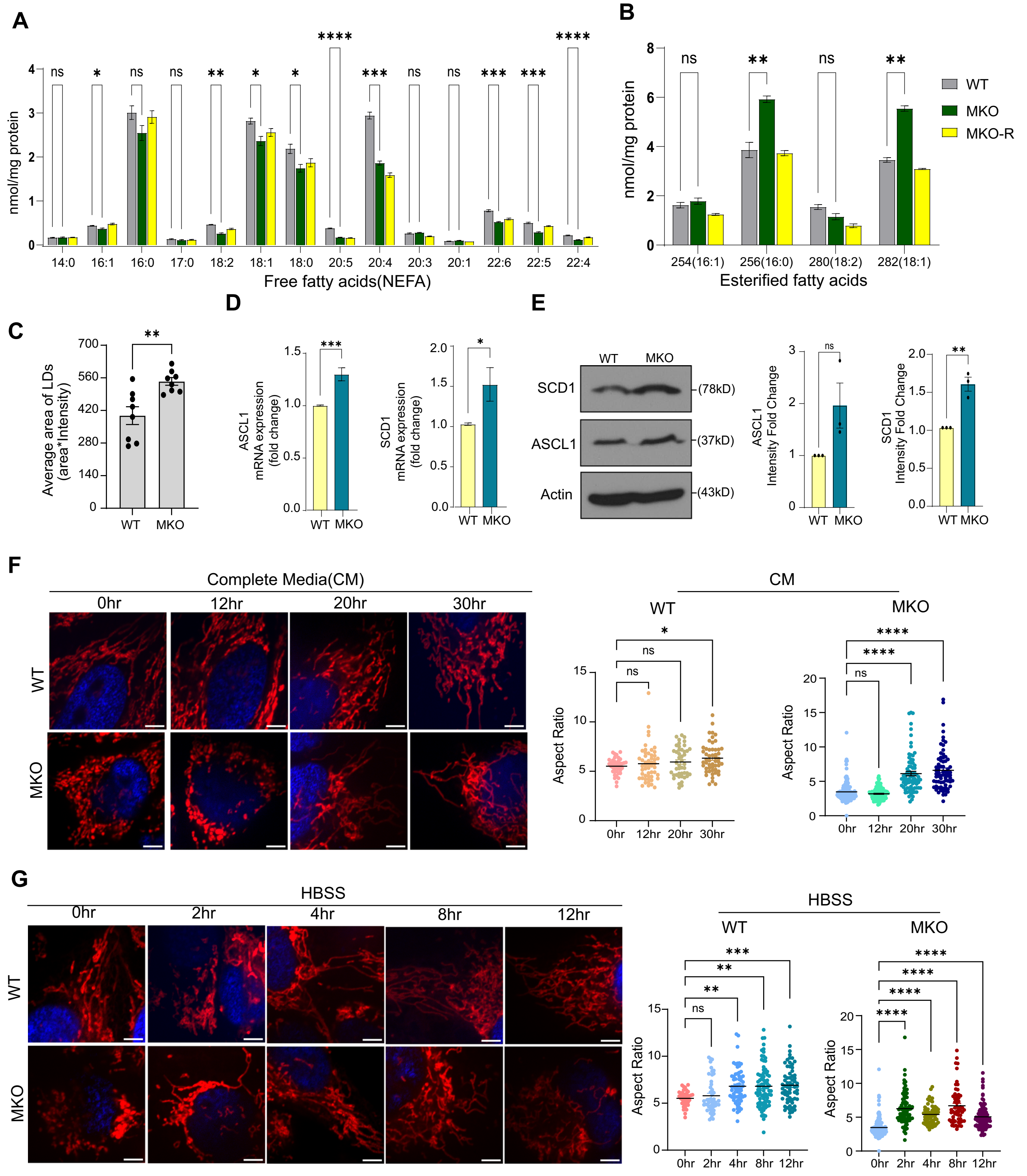

A
nmol/mg protein
14:0 16:1 16:0 17:0 18:2 18:1 18:0 20:5 20:4 20:3 20:1 22:6 22:5 22:4
Free fatty acids(NEFA)
B
nmol/mg protein
WT
MKO
MKO-R
254(16:1) 256(16:0) 280(18:2) 282(18:1)
Esterified fatty acids
C
Average area of LDs (area*Intensity)
WT MKO
D
ASCL1 mRNA expression (fold change)
SCD1 mRNA expression (fold change)
WT MKO
E
WT MKO
SCD1 (78kD)
ASCL1 (37kD)
Actin (43kD)
ASCL1 Intensity Fold Change
SCD1 Intensity Fold Change
F
Complete Media(CM)
0hr 12hr 20hr 30hr
WT
MKO
CM
Aspect Ratio
G
HBSS
0hr 2hr 4hr 8hr 12hr
WT
MKO
Aspect Ratio

© The Author(s)

◀ **Figure EV4. MKO cells show accelerated mitochondria elongation under nutrient depletion conditions.**

(**A**, **B**) The levels of Free fatty acids (NEFA)(A) and Esterified fatty acids (**B**) in all 3 cell lines. Results are presented as mean ± SEM (ns, non-significant, *P<0.05, **P<0.001, ***P<0.0003, ****P<0.0007; one-way ANOVA, $n = 4$ independent biological replicates). (**C**) Quantification of LD average size in WT and MKO cells. The average LD size from different time points was combined and plotted as a single group for both WT and KO cells. Results are presented as mean ± SEM (**P<0.001; Unpaired t test, n=3 independent biological replicates). (**D**) mRNA levels of ACSL1 and SCD1in WT and MKO cells. Results are presented as mean ± SEM (*P<0.05, ***P<0.0003; Unpaired t test, $N = 3$ independent experiments). (**E**) Left panel: Western blot for ACSL1 and SCD1 proteins in lysates from WT and MKO cells 20 h-post media change. Right panels: Quantification of relative density of ACSL1 and SCD1 normalized to Actin (loading control). Results are presented as mean ± SEM (ns, non-significant, **P<0.001, Unpaired t test). $N = 3$ independent experiments (**F**). Analyses of mitochondria morphology. Left panel: WT and MKO cells were plated into complete media (CM), then media was refreshed (considered as time 0) and pictures were taken at 0, 12, 20, and 30 h-post media change. Mitochondria were labeled using Mito Tracker Deep red (MTDR). Scale bar=5 μm. Right panels: Quantification of mitochondria morphology of WT and MKO cells. Results are presented as mean ± SEM (ns, non-significant, *P<0.05, ****P<0.0007; Unpaired t-test, N=3 independent experiments). (**G**) Left panel: Analyses of mitochondria morphology in WT and MKO cells incubated in HBSS, and pictures were taken at 2, 4, 8 and 12-h post incubation and labeled as in (**F**). Scale bar = 5 μm. Right panels: Quantification of mitochondria morphology of WT and MKO cells. Results are presented as means ± SEM (ns, non-significant, **P<0.001, ***P<0.0003, ****P<0.0007; $n = 3$ Independent biological replicates). Source data are available online for this figure.

 © The Author(s)

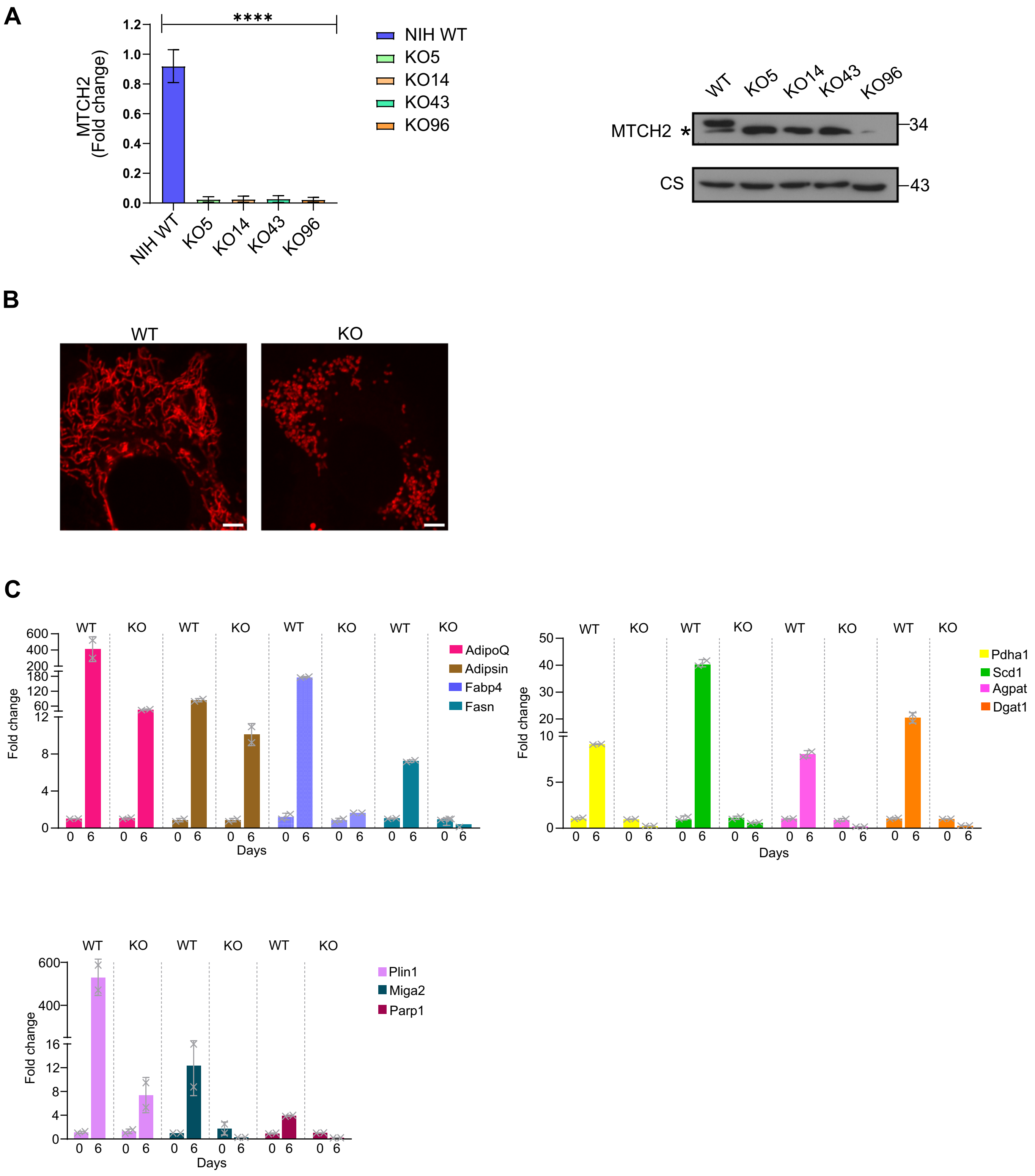
A
MTCH2 (Fold change)
****
NIH WT
KO5
KO14
KO43
KO96
WT
MTCH2
CS
34
43
B
WT
KO
C
Fold change
Days
AdipoQ
Adipsin
Fabp4
Fasn
Pdha1
Scd1
Agpat
Dgat1
Plin1
Miga2
Parp1

Figure EV5. MTCH2 is critical for adipocyte differentiation.

(**A**) MTCH2 mRNA expression was checked by RT-PCR (left panel) and protein level were checked by Immunoblot (right panel) in 4 different MTCH2 knockout (KO) and WT clones. *, nonspecific band. Results are presented as mean ± SEM of one representative out of three independent experiments (****P<0.0007, ordinary one-way ANOVA). (**B**) Mitochondrial morphology in NIH3T3L1 Preadipocytes. MTCH2 knockout (KO) leads to mitochondrial fragmentation. Mitochondria were labeled using MitoTracker Deep red (MTDR). Scale bar = 5 µm. (**C**) RT-PCR of WT and MTCH2 knockout (KO) cells at day 0 and day 6-post differentiation. Components of the adipogenic effector genes were analyzed: adiponectin (AdipoQ), Adipsin, fatty-acid-binding protein 4 (Fabp4), fatty acid synthase (FASN), pyruvate dehydrogenase (Pdha1), stearyl-CoA desaturase (Scd1), 1-acyl-sn-glycerol-3-phosphate (Agpat), diacylglycerolacyltransferase (Dgat1), perilipin (Plin1), mitoguardin 2 (Miga2), and poly(ADP-ribose) polymerase1 (Parp1). Results are presented as mean ± SD of one representative out of three independent experiments. Normalization was done by taking geometric mean of three housekeeping genes, Importin, Tubulin and AcTH. Source data are available online for this figure.

 © The Author(s)
