# Supplementary material for: MTCH2 controls energy demand and expenditure to fuel anabolism during adipogenesis
Source: EMBO J. 2025 Jan 3;44(4):1007–38. doi: 10.1038/s44318-024-00335-7 (PMC11832942; doi:10.1038/s44318-024-00335-7)
Supplement: Supplementary file 10 — EV Figure Source Data [file 44318_2024_335_MOESM10_ESM.zip › Figure_EV1.zip!/Figure EV1/Figure EV1_B.pdf]

Figure EV1_B

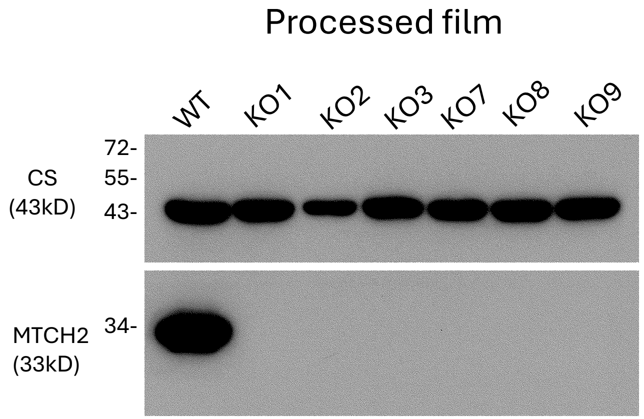

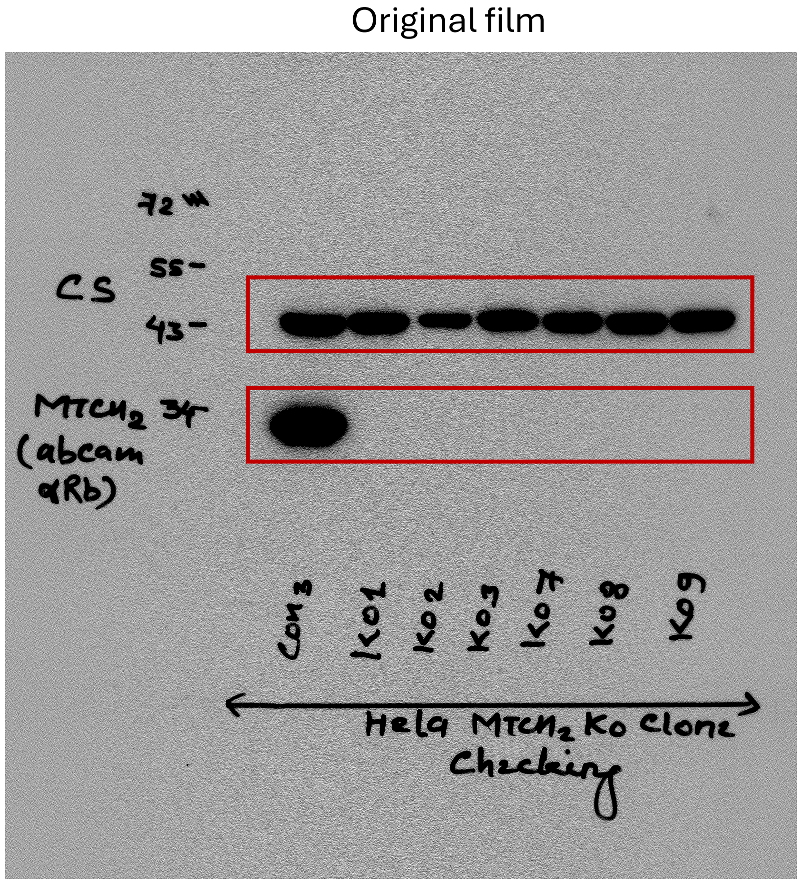

A representative blot is shown here, used for figure preparation. The membrane was cut and probed with antibodies for MTCH2 (human) and Citrate Synthase (CS), then developed on the same X-ray film. The red box highlights the area selected for the figure.
