# Supplementary material for: MTCH2 controls energy demand and expenditure to fuel anabolism during adipogenesis
Source: EMBO J. 2025 Jan 3;44(4):1007–38. doi: 10.1038/s44318-024-00335-7 (PMC11832942; doi:10.1038/s44318-024-00335-7)
Supplement: Supplementary file 10 — EV Figure Source Data [file 44318_2024_335_MOESM10_ESM.zip › Figure_EV4.zip!/Figure EV4/Figure EV4_E.pdf]

Figure EV4_E

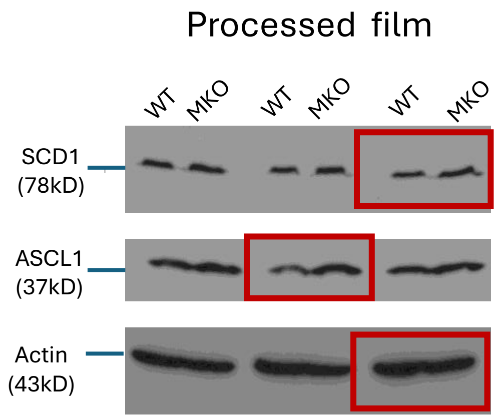

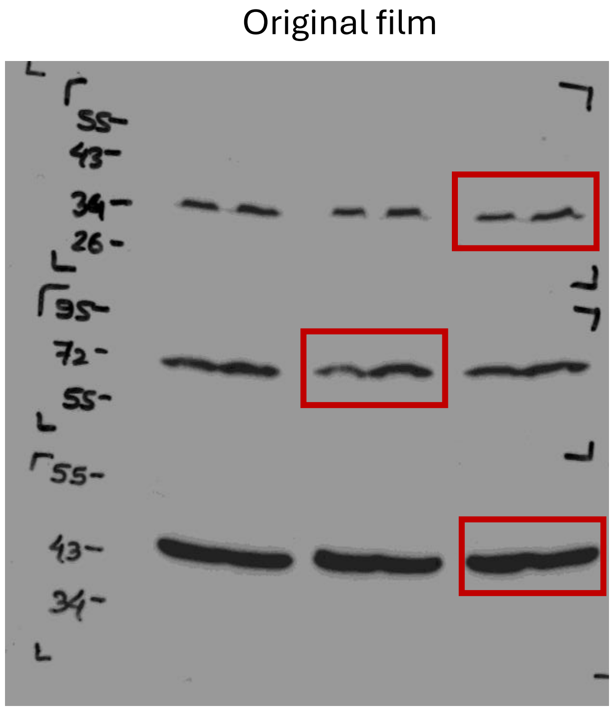

Blots from three independent experiments were performed. The membrane was cut and probed with antibodies for SCD1, ASCL1, and Actin, and developed on the same X-ray film. The red box indicates the representative area selected for figure preparation.
