# Supplementary material for: MTCH2 controls energy demand and expenditure to fuel anabolism during adipogenesis
Source: EMBO J. 2025 Jan 3;44(4):1007–38. doi: 10.1038/s44318-024-00335-7 (PMC11832942; doi:10.1038/s44318-024-00335-7)
Supplement: Supplementary file 10 — EV Figure Source Data [file 44318_2024_335_MOESM10_ESM.zip › Figure_EV5.zip!/Figure EV5/Figure EV5_A .pdf]

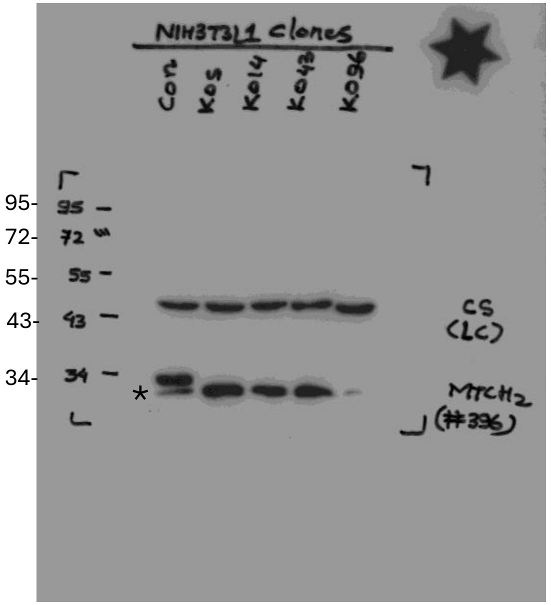

* =nonspecific band

Membrane were cut and probe with MTCH2(mouse) and Citrate Synthase(CS) and then develop on the x-ray film
